# CRTC1 transcriptional coactivator is required for hepatitis B virus gene expression and replication

**DOI:** 10.1186/2049-3002-2-S1-P31

**Published:** 2014-05-28

**Authors:** Hei-Man Vincent Tang, Wei-Wei Gao, Chi-Ping Chan, Kin-Hang Kok, Dong-Yan Jin

**Affiliations:** 1 Department of Biochemistry and State Key Laboratory for Liver Research, The University of Hong Kong, Pokfulam, Hong Kong

## Background

Chronic hepatitis B virus (HBV) infection occurs in over 400 million people worldwide, 15-40% of whom will terminally develop severe liver diseases including hepatocellular carcinoma. Although development of HCC is a multi-step process, high HBV DNA level is a major risk factor for disease progression. Transcription of HBV from the cccDNA template is essential for its replication and requires CREB transcription factor, a master regulator of cell metabolism. However, transcriptional coactivators that facilitate CREB-dependent activation of HBV transcription remain to be identified and characterized.

## Results

In this study we demonstrate that cellular CRTC transcriptional coactivators, which also play important roles in liver metabolism, are required for HBV transcription and replication. CRTC1 expression was elevated in HBV-positive hepatoma cells and liver tissues. Ectopic expression of CRTC1 in HepG2 hepatoma cells stimulated preS2/S promoter activity of HBV, whereas overexpression of a dominant inactive form of CRTC1 resulted in suppression of activity. CRTC1 interacts with CREB and they are mutually required for the recruitment to preS2/S promoter and the activation of HBV transcription. The expression of pregenomic RNA (pgRNA) and the level of cccDNA were upregulated when CRTC1 was overexpressed, whereas the levels of pgRNA, cccDNA and HBsAg were diminished when CRTC1 was compromised. In addition, HBV transactivator protein HBx stabilized CRTC1 and promoted its activity on HBV transcription.

## Conclusion

Our work reveals an essential role of CRTC1 in HBV transcription and an unrecognized link between liver metabolism and HBV oncogenesis mediated through a shared transcriptional coactivator.
